# Race, Wealth, and Solid Waste Facilities in North Carolina

**DOI:** 10.1289/ehp.10161

**Published:** 2007-07-09

**Authors:** Jennifer M. Norton, Steve Wing, Hester J. Lipscomb, Jay S. Kaufman, Stephen W. Marshall, Altha J. Cravey

**Affiliations:** 1 Department of Epidemiology, University of North Carolina at Chapel Hill, Chapel Hill, North Carolina, USA; 2 Division of Occupational and Environmental Medicine, Department of Community and Family Medicine, Duke University Medical Center, Durham, North Carolina, USA; 3 Department of Geography, University of North Carolina at Chapel Hill, Chapel Hill, North Carolina, USA

**Keywords:** environmental health, environmental justice, solid waste

## Abstract

**Background:**

Concern has been expressed in North Carolina that solid waste facilities may be disproportionately located in poor communities and in communities of color, that this represents an environmental injustice, and that solid waste facilities negatively impact the health of host communities.

**Objective:**

Our goal in this study was to conduct a statewide analysis of the location of solid waste facilities in relation to community race and wealth.

**Methods:**

We used census block groups to obtain racial and economic characteristics, and information on solid waste facilities was abstracted from solid waste facility permit records. We used logistic regression to compute prevalence odds ratios for 2003, and Cox regression to compute hazard ratios of facilities issued permits between 1990 and 2003.

**Results:**

The adjusted prevalence odds of a solid waste facility was 2.8 times greater in block groups with ≥50% people of color compared with block groups with < 10% people of color, and 1.5 times greater in block groups with median house values < $60,000 compared with block groups with median house values ≥$100,000. Among block groups that did not have a previously permitted solid waste facility, the adjusted hazard of a new permitted facility was 2.7 times higher in block groups with ≥50% people of color compared with block groups with < 10% people of color.

**Conclusion:**

Solid waste facilities present numerous public health concerns. In North Carolina solid waste facilities are disproportionately located in communities of color and low wealth. In the absence of action to promote environmental justice, the continued need for new facilities could exacerbate this environmental injustice.

Disposition of solid waste is an ancient public health problem made more pressing in the 21st century by population growth, increases in per capita waste production, scarcity of suitable locations for waste disposal near cities, and changes in the composition of waste (Melosi 2005). In recent decades there have been environmental health concerns about contamination of increasingly scarce potable water sources by landfill leachate and air pollution from landfill gases [Agency for Toxic Substances and Disease Registry (ATSDR) 2001]. These have led to requirements that municipal solid waste landfills (MSWLs) use plastic liners and install leachate and gas collection systems [U.S. Environmental Protection Agency (EPA) 1993]. These systems should reduce and delay off-site environmental impacts of MSWLs; however, some other solid waste facilities, notably construction and demolition landfills, do not require liners or gas collection.

Choosing locations for MSWLs and other solid waste facilities involves consideration of local geology and hydrology, existing land uses, proximity to waste sources, and transportation routes. As requirements for expensive engineering controls have been implemented, economies of scale have led to a decline in the number of active solid waste disposal sites along with an increase in their average size (U.S. EPA 2002). Larger facilities that will be used for longer time periods have greater potential to impact other local land uses and neighboring populations. Obtaining permits for new solid waste facilities has become increasingly difficult because of stricter environmental requirements and opposition from local communities (U.S. EPA 1993). Some communities have questioned whether they should host the waste produced by distant populations (Bullard 2000). Solid waste disposal, like many other practices such as treatment and disposal of hazardous wastes, oil refining, chemical production, and industrial animal agriculture, may threaten environmental health conditions in host communities so that other communities can reap the benefits of production without suffering the most direct environmental consequences. A disproportionate burden of such facilities or pollutants in poor communities and communities of color is often referred to as “environmental injustice.” With few exceptions (Faber and Krieg 2002; General Accounting Office 1995), environmental injustice concerns related to solid waste facilities in the United States have not been examined.

In North Carolina, recent proposals to build landfills have generated concerns that the state will become a major importer of wastes produced in other states (Henderson 2005; Rawlins 2005). Several permit applications from private companies that want to operate large regional landfills in eastern North Carolina, a poor and historically African-American region of the state, were under consideration when the state legislature adopted a 1-year moratorium on new landfills in July 2006 (General Assembly of North Carolina 2006). The North Carolina Environmental Justice Network, a coalition of community-based organizations, called for a moratorium on construction of new landfills in poor communities and communities of color until wealthy communities accepted their share of waste (North Carolina Environmental Justice Network 2003). However, the location of existing solid waste facilities in relation to community race and wealth has not been evaluated previously.

This study was conducted to evaluate two environmental justice questions with implications for environmental health and health disparities. First, we examined records for solid waste facilities present in 2003 to determine whether they are disproportionately located in communities of color and in poor communities. This cross-sectional analysis, however, could not determine whether these communities are more often selected for landfills, or whether the race and wealth of communities changed after the facilities were built. Therefore, longitudinal analyses were also conducted of the facilities that received permits between 1990 and 2003 to determine the race and wealth of the communities at the time the facilities received permits.

## Materials and Methods

We used inhabited North Carolina census block groups as the unit of analysis to define communities in order to obtain racial and economic characteristics (*n* = 5,261). Census block groups are designed to contain between 600 and 3,000 people, with an optimum size of 1,500 people (U.S. Census Bureau 2001b). The choice of block group boundaries is intended, where possible, to reflect neighborhoods with distinct sociodemographic characteristics.

### Solid waste facilities

The North Carolina Division of Waste Management (NCDWM) is responsible for issuing permits to solid waste facilities in the state. The NCDWM’s electronic files of permitted solid waste facilities lacked sufficient information, including dates of operation and specific facility locations, to address the study aims. Thus, we obtained additional information through a systematic review of solid waste facility paper records maintained by the NCDWM. For the purpose of this study, solid waste facilities were defined as MSWLs, construction and demolition debris landfills (CDLFs), industrial solid waste landfills (INDUSLFs), tire landfills (TIRELFs), and waste transfer stations (TRANSFERs). Solid waste facilities that were issued a permit to operate (or equivalent) by 31 December 2003 were included in the study.

We used geographic coordinates to locate solid waste facilities within block groups. The North Carolina Center for Geographic Information Analysis (NCCGIA) provided geographic coordinates for some solid waste facilities. Complete addresses were not available for most solid waste facilities; therefore, address matching could not be used to locate facilities. We used maps obtained from NCDWM records, tax parcel maps, and the internet program TerraFly (Florida International University 2003) to determine latitudes and longitudes and to verify coordinates received from the NCDWM and the NCC-GIA. The TerraFly interface was used to virtually fly over aerial images to locate solid waste facilities. When possible, latitudes and longitudes at the approximate centers of waste disposal areas or TRANSFER buildings were recorded. The coordinates of two solid waste facilities that could not be visually located were assigned to the centroid coordinates of the census block group that contained the road listed as the facility address. Census 2000 geographic boundary files were obtained from ESRI (2001). Geographic coordinates of solid waste facilities were spatially joined to census block groups using ArcGIS version 9.1 (ESRI, Redlands, CA).

### Block group characteristics

We obtained block group population and wealth data from the U.S. Census Bureau decennial Census 2000 Summary File 3 (U.S. Census Bureau 2002a) and the 1990 Census Summary File 3 (GeoLytics Inc. 2003). Census 2000 data were used for analyses of existing facilities in 2003. For analyses of newly permitted facilities after 1990, we used a standard geographic area to compute changes over time so that changes in the population are not a reflection of changing geographic boundaries. The longitudinal analyses utilized data from GeoLytics, which estimates 1990 U.S. Census data in 2000 Census block group boundaries (GeoLytics Inc. 2003). Intercensal estimates were created by linear interpolation between 1990 and 2000. Linear extrapolation beyond the year 2000 yielded implausible values from some areas; therefore, we used year 2000 data to estimate the population for 2001–2003 for each block group.

The primary racial and ethnic groups in North Carolina are white non-Hispanic (70%), African-American non-Hispanic (21%), Hispanic (5%), Asian non-Hispanic (1%), and American Indian non-Hispanic (1%) (U.S. Census Bureau 2002a). Racial and ethnic groups other than white non-Hispanic share experiences of discrimination, low political power, and low socioeconomic status (Smelser et al. 2001). Hispanic identity is an ethnic, not a racial, classification. North Carolina’s Hispanic population is largely made up of recent immigrants from Mexico and Central America (U.S. Census Bureau 2001a). Because of these shared characteristics and small population size of racial and ethnic groups other than white non-Hispanic and African American, race was categorized as either white non-Hispanic or other, and the percentage of persons of race and ethnicity other than white non-Hispanic was calculated for each block group.

Socioeconomic status may be measured by education, annual income, or wealth. Wealth varies less over time than annual income and is a primary dimension of social class and political power in the United States (Krieger et al. 1997). We used median house value for owner-occupied housing units as a measure of community wealth. For the cross-sectional analyses of facilities present in 2003, all owner-occupied housing units were used. For the longitudinal analyses of facilities permitted between 1990 and 2003, data were available only from the 1990 Census for specified owner-occupied housing units. Specified housing units do not include mobile homes, homes with a business on the property, or homes on > 10 acres of land (U.S. Census Bureau 2002b). Median house values in 1990 were adjusted to year 2000 dollars to account for inflation (U.S. Census Bureau 2004).

Several other factors were considered as alternative explanations of solid waste facilities. Landfills require land for waste disposal, which is more plentiful in rural areas. Many rural areas of North Carolina have high poverty levels, and eastern North Carolina is part of the Black Belt, the former slave plantation region of the South that is still home to many rural African Americans. Population density, expressed as persons per square mile, was used to measure rurality. We also considered region of the state as a determinant of locations of solid waste facilities. North Carolina has four regions that differ physically, racially, and economically: Mountain, Piedmont, Coastal Plain, and Tidewater (Gade et al. 1986). Because access to truck routes connecting points of waste generation to points of waste disposal is considered in locating solid waste sites, we also considered block group distance to the nearest major road and distance to the nearest city [defined by the U.S. Census Bureau (2001b) as “urbanized area/urban cluster”] measured from block group centroids as a determinant of the location of solid waste facilities.

### Statistical analysis

We used inhabited census block groups as the unit of analysis. For cross-sectional analyses, the outcome was whether or not the block group had ≥1 permitted solid waste facility on 31 December 2003. Crude and adjusted prevalence odds ratios (PORs) and 95% confidence intervals (CIs) were calculated using logistic regression with generalized estimating equations to account for the nesting of block groups within counties. Models for all facility types and for specific facility types were fit and indicator variables were created to compare block groups with higher percentages of people of color to those with lower percentages of people of color (< 10%), and to compare block groups with lower house values block groups to those with the highest house values (≥$100,000). The combined effects of race and house value were assessed by creating indicator variables for their cross-classification. Population density was modeled as a continuous variable. Region was considered using indicator variables with the Piedmont as the referent category. We considered distance ≥3 miles from a major population center in North Carolina, using an indicator variable with block groups < 3 miles from a city as the referent category. Indicator variables were also used to evaluate distances from major roads using the following categories: block groups ≥1 mile from a U.S. highway and ≥10 miles from an interstate; block groups < 1 mile from a U.S. highway or < 10 miles from an interstate; and block groups < 1 mile from a U.S. highway and < 10 miles from an interstate (referent).

Longitudinal analyses followed block groups through time between 1990 and 2003. Because the presence of an existing solid waste facility strongly affects permitting of another facility at the same location, facility-free block groups were analyzed separately from block groups with an existing facility. We used a block group-time (analogous to person-time) approach so that block groups contributed follow-up time in each category of race and wealth as they changed over time; block groups remained at risk of having a first facility permitted (for facility-free block groups) or for having an additional facility permitted (for block groups with existing facilities). Facility-free block groups entered follow-up in the cohort with existing facilities on the date of their first permitted facility, at which time they were at risk of receiving a second permitted facility. Crude and adjusted hazard ratios (HRs) were calculated using extended Cox proportional hazards regression to compare block groups with higher percentages of people of color and lower house values to block groups with lower percentages of people of color and higher house values. Because of the nesting of block groups within counties, SEs were computed using robust variance estimation.

## Results

### Characteristics of solid waste facilities

Permit records were reviewed and electronically recorded for 536 facilities. Ninety-three facilities were excluded because they were not classified as an eligible facility type; 24 other facilities were excluded because they received a permit to operate after the end of the study period (*n* = 8) or because they had not been constructed by the end of the study period (*n* = 16). Therefore, 419 solid waste facilities were eligible to be included in the study.

The number, type, operation status, permit period, and owner/operator of these 419 solid waste facilities are provided in Table 1. MSWLs comprise the largest solid waste facility category (48%), followed by TRANSFERs (22%), CDLFs (18%), INDUSLFs (12%), and TIRELFs (1%). There were 194 facilities open to accept waste for disposal or transfer on 31 December 2003. TRANSFERs had the largest proportion of open facilities (86%), followed by CDLFs (84%), TIRELFs (67%), INDUSLFs (21%), and MSWLs (20%). Permits were issued to 207 solid waste facilities to construct and/or operate after 1 January 1990. TRANSFERs comprise the largest category of solid waste facilities issued permits during this period (42%) followed by CDLFs (35%), MSWLs (19%), INDUSLFs (3%), and TIRELFs (< 1%).

MSWLs were widely distributed in North Carolina, located in 97 of 100 counties; 251 block groups (4.8%) contained at least one solid waste facility. MSWLs were present in 3.2% of block groups, TRANSFERs in 1.6%, CDLFs in 1.4%, and INDUSLFs in 0.8%.

### Race and wealth of block groups

**Figure 1:**
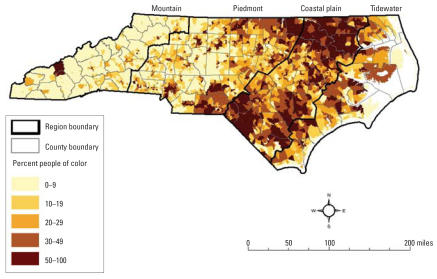
Percentage of people of color in the population: North Carolina block groups, 2000.

The spatial distribution of race in 2000 is shown in Figure 1. The highest percentages of populations of color, primarily African Americans, are in the Coastal Plain and in the large Piedmont cities of Charlotte, Winston-Salem, Greensboro, Durham, and Raleigh. In the Mountain region, many American Indians reside on and near the Cherokee Indian Reservation in Swain and Jackson Counties. The population of Robeson County, at the southern border of the Coastal Plain, is roughly evenly divided between African American, American Indian, and white.

**Figure 2:**
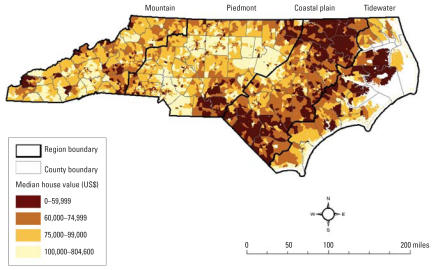
Median house value of all owner-occupied housing units: North Carolina block groups, 2000.

The spatial distribution of house values is shown in Figure 2. Housing values are highest in the Piedmont, with notably high values in some areas of the Mountain and Tidewater regions. The lowest housing values are in the Coastal Plain.

### Cross-sectional analyses

We quantified relationships of race and house value with solid waste facility locations, with adjustment for other predictors of facility location. The presence of one or more permitted solid waste facilities in 2003 showed a strong inverse relationship with population density. This was modeled using a cubic polynomial for the natural log of population density (the likelihood ratio test for addition of these terms to an intercept-only logistic model was 190.9; 2 degrees of freedom). Additional polynomial terms did not contribute substantially to model fit. The prevalence of any solid waste facility was highest in the Tidewater region (8.4% of 513 block groups), followed by the Mountain region (6.5% of 835 block groups), Coastal Plain (4.4% of 1,087 block groups), and the Piedmont (3.8% of 2,826 block groups). Compared with the Piedmont, the prevalence odds of any solid waste facility, adjusted for population density, were 1.8 (95% CI, 1.2–2.7) times higher in the Tidewater region. Adjusted PORs were slightly higher for the Mountain region (POR = 1.1; 95% CI, 0.8–1.6) and slightly lower for the Coastal Plain (POR = 0.9; 95% CI, 0.6–1.2), compared with the Piedmont. After adjustment for population density, distances from urban areas and major roads were not associated with prevalence of solid waste facilities. These variables did not affect estimated relationships of waste facility locations with race and house value and therefore were not included in subsequent models.

Table 2 provides adjusted PORs for solid waste facility types by race and house value. Results are not provided separately for TIRELFs or INDUSLFs because of small numbers. The adjusted PORs of any solid waste facility, and of each facility type, were approximately 2–3 times higher in block groups with ≥20% people of color compared with block groups with < 10% people of color. Adjusted PORs in block groups with 10 to < 20% people of color were between unity and 2, compared with block groups with < 10% people of color. Table 2 also provides summary values for block groups with ≥10% people of color compared with block groups with < 10%; these ranged from 2.1 for any solid waste facility to 2.5 for TRANSFERs.

Compared with block groups with median house values > $100,000, adjusted PORs ranged from 1.2 to 1.8 for any solid waste facility and any MSWL. Adjusted PORs were less than unity for construction and demolition landfills, and between 0.8 and 1.5 for TRANSFERs. Summary PORs comparing block groups with median house values < $100,000 with those with greater values ranged from 0.9 for construction and demolition landfills to 1.5 for MSWLs.

Adjusted PORs for cross-classified levels of race and house value are presented in Table 3. The prevalence odds of any solid waste facility increased as median house value decreased among white block groups. Among high-wealth (≥$100,000) and medium-wealth ($60,000 to < $100,000) block groups, the prevalence odds of any solid waste facility increased as the percentage of people of color in the population increased. PORs ranged between 3.0 and 5.6 for block groups with ≥10% people of color and house values < $100,000, and for block groups with < 10% people of color and house values < $60,000.

### Longitudinal analysis

Of the block groups that received at least one permitted solid waste facility between 1990 and 2003, most (93/146) also contained at least one solid waste facility permitted before 1 January 1990. To account for the difference in the baseline risk of new permitted solid waste facilities, we conducted a stratified analysis based on the presence of any previously permitted solid waste facility in the block group.

Table 4 provides adjusted HRs for solid waste facilities newly permitted between 1990 and 2003 by race and house value, stratified by the presence of any previously permitted solid waste facility. Because of small numbers, results are not presented separately by facility type. Among block groups that did not have a permitted solid waste facility eligible to be included in the study before 1 January 1990, the hazards of any new solid waste facility were 1.6–3.0 times higher in block groups with ≥10% people of color compared with block groups with < 10% people of color. Among block groups that contained a previously permitted solid waste facility eligible to be included in the study, race was not strongly associated with the hazard of any new solid waste facility (HRs ranged from 0.8 to 1.1).

Among block groups that did not have a previously permitted solid waste facility (compared with block groups with median house values ≥$100,000), adjusted HRs for medium wealth block groups, median house values $60,000 to < $75,000 and $75,000 to < $100,000, respectively, were 0.6 and 0.5 times as high. Among block groups with a previously permitted solid waste facility (compared with block groups with median house values ≥$100,000), adjusted HRs for medium wealth block groups (median house values $60,000 to < $75,000 and $75,000 to < $100,000, respectively) were 1.3 and 1.4 times higher.

Table 5 presents adjusted HRs by owner/operator of the first solid waste facility newly permitted between 1990–2003. Privately owned and/or operated facilities were permitted at a 2.4 times higher rate in block groups with ≥10% people of color, compared with block groups with < 10%. Permitting of publicly owned and operated solid waste facilities was not related to race (HR = 1.0). Compared with block groups with median house values ≥$100,000, the hazard of any new solid waste facility in block groups with median house values < $100,000 was similar for private and public facilities (HR = 0.9 and 0.8, respectively).

## Discussion

This is the first study to examine the statewide location of permitted solid waste facilities in North Carolina, and one of the few studies of environmental injustice and solid waste facilities. We found that, accounting for population density and region, the prevalence odds of a solid waste facility in 2003 were greater in North Carolina block groups with larger proportions of people of color compared with white block groups, and greater in lower wealth block groups compared with high wealth block groups. We also found that in block groups without solid waste facilities, adjusting for population density, during 1990–2003 new facilities were permitted at a higher rate in block groups with larger proportions of people of color compared with block groups with < 10% people of color. This relationship was observed for private but not public facilities.

Our results are consistent with a statewide analysis conducted in Massachusetts by Faber and Krieg (2002). These authors evaluated the location of solid waste landfills and TRANSFERs in relation to race and income of towns as part of an analysis of cumulative exposures to ecologic hazards. They reported higher concentrations of these facilities among nonwhite and lower-income communities compared with white and higher-income communities.

We acknowledge several limitations to the present study. We could not examine all types of solid waste facilities. Although most major types of facilities were included, different location patterns may exist for other facility types, such as land-clearing and inert debris landfills and preregulatory dumps. Furthermore, we did not count permits issued to existing facilities that served to expand the amount of waste disposed or increase the waste service area. A final limitation concerns the nature of facility location data. Using a point on a map to represent solid waste facilities could lead to mis-classification of which block groups contain facilities. The method used to obtain and verify coordinates was more sensitive to correctly identifying the block groups that contained the waste disposal area or transfer station building rather than block groups that contained the facility entrance, when these block groups are different. We conducted a pilot test for 52 solid waste facilities to compare the coordinates we obtained from TerraFly, based on the waste disposal area, to the coordinates available from the NCCGIA, which were reported to be taken at the facility entrance. In this pilot test, block group assignment differed between these methods for only one facility. This represented an extreme example where the facility gate entrance was located in a different county than the waste disposal area.

The present study also had a number of strengths. Longitudinal analyses of new facilities were conducted separately for areas with and without solid waste facilities, because an existing solid waste facility is the most important determinant of the location being selected for a new facility during this time period. For example, between 1990 and 2003, 81% of the newly permitted CDLFs, 69% of the lined MSWLs, and 53% of the TRANSFERs were permitted in the 153 block groups with an existing unlined MSWL. This provides strong support for the hypothesis that existing landfills attract additional solid waste facilities. This effect means that the burden of solid waste on people of color will be difficult to reverse without addressing the momentum created by the historical pattern of disproportionate siting of solid waste facilities in areas with more people of color.

Our finding that newly permitted solid waste facilities and race were related only in areas that were previously free of facilities suggests that areas with solid waste facilities in 1990 did not attract additional facilities due to race because these areas already had higher percentages of persons of color, resulting in less variation in race in areas with previously permitted facilities, so race cannot be as predictive. Another explanation for this finding could be that these block groups could not support the addition of another facility because of the saturation of land uses, a factor that we did not measure in this study. For example, in 1990, the prevalence odds of having a solid waste facility, adjusted for population density and region, were 2.0 in block groups with > 10% persons of color compared with block groups with < 10% persons of color.

Most solid waste facilities are publicly owned and operated by local governments, reflecting the 20th century practice of managing solid waste as a public good (Pinch 1985). Nearly all of the INDUSLFs are privately owned and operated, reflecting the use of these facilities for industrial solid wastes generated through manufacturing processes. More recently, the vertical integration of the waste management industry has resulted in privately owned and operated solid waste facilities or public–private partnerships. As costs of landfill construction have risen, the number of new facilities has declined while the size of new facilities has increased. Unlike municipalities, private waste management companies have not commonly owned solid waste facilities; thus, they may need to seek new, more remote locations that can accommodate facilities that serve large regions. These trends would be consistent with our observation that relationships between newly permitted facilities and race were observed only for privately owned and/or operated facilities.

### Environmental injustice, solid waste, and health

Environmental injustice and solid waste are public health issues. Proper solid waste management has long been a public health concern. Many facilities that were formerly used for municipal solid waste disposal are now a source of groundwater contamination (North Carolina Division of Waste Management 2003; U.S. EPA 1993). Landfills are also a source of odorous and nonodorous gases (ATSDR 2001). One mechanism through which landfills can affect health is through direct exposure to harmful toxicants found in landfill gases. Several epidemiologic studies have evaluated this hypothesized pathway using residential proximity to landfills as a proxy for exposure. The results of these studies suggest that living near MSWLs is associated with elevated risks of poor birth outcomes including low birth weight (Elliott et al. 2001; Goldberg et al. 1995b); respiratory conditions including bronchitis and shortness of breath (Hertzman et al. 1987); site-specific cancers of the stomach, liver, and pancreas (Goldberg et al. 1995a, 1999); and experience of malodors (Berger et al. 2000).

Another mechanism through which solid waste landfills can affect health is through the built environment (i.e., buildings, open areas, and infrastructure created and maintained by human action). Odor, noise, traffic, and visual pollution from landfills may act as repellents to health-promoting amenities in communities, such as health clinics, food stores, and recreational facilities, which could adversely affect access to medical care, diet, and physical activity. Residents living in close proximity to active landfills and TRANSFERs may be impacted directly by noise exposures from daily activities at the facility. Noise exposures can affect well-being and induce stress (Passchier-Vermeer and Passchier 2000). Heavy truck traffic on roads leading to solid waste facilities may present safety concerns (Eyles et al. 1993).

People of color and low-wealth populations may be more vulnerable to specific environmental agents as well as problems of the built environment created by solid waste facilities. Examples of predisposing factors that could increase individual susceptibility in communities of color and those of low-income are young or old age, higher disease prevalence, less access to nutritious foods, and increased exposure to occupational hazards. Factors that could promote exposures include unprotected drinking water sources, poorly insulated housing, limited access to transportation, and lack of money for household protection (any measures that could be used to seal houses and minimize potential exposures). Community vulnerability may be due to factors that increase individual susceptibility, promote individual exposure, or limit the collective ability of communities to prevent or ameliorate negative impacts of waste facilities. Collective wealth and political influence can provide communities with resources for preventing the siting of facilities, improving the built environment when it is threatened, and implementing bigger buffers, better engineering, and better management practices when facilities are sited. Thus, communities of color and poor communities would tend to experience greater health impacts from a solid waste facility than communities with greater resources. Any negative impacts of solid waste facilities on the health of neighboring communities in North Carolina might be lessened by preventing them from being located disproportionately in the most susceptible areas.

Malodor from landfill gases may create barriers to siting health-promoting facilities such as food stores, parks, sports facilities, and walking trails. Even where health-promoting facilities exist near landfills, actual and anticipated malodors may limit participation in outdoor physical activity. In a recent survey of 267 middle schools in North Carolina, Mirabelli (2005) found that staff at 23 schools reported landfill odors on school grounds and at 7 reported landfill odors inside the schools.

Production of solid waste in the United States has been increasing over the entire period for which production estimates are available (U.S. EPA 2005). Increases have been observed on both a per capita and total basis. Although we focused on North Carolina, the results have national and international implications. Solid waste and related disposal problems can be alleviated by reducing waste, and reuse and recycling. Although waste reduction policies, including zero waste initiatives, have been proposed, there is little incentive for waste reduction when waste-producing communities are not regularly exposed to the solid waste facilities that are the inevitable consequences of their production. Waste production is related to consumption of goods (U.S. EPA 2006). In North Carolina, wealthy and white populations have a paucity of disposal facilities. Table 3 shows that adjusted PORs for areas with > 10% people of color and house values < $100,000 vary from 3.1 to 4.1. The ability of populations that produce the most waste to dispose of the waste in areas that lack resources and political power increases the potential for disparate impacts on public health and also eliminates the feedback between production and consumption that could create pressure to reduce the amounts of waste produced. Environmental injustice in the locations of solid waste sites therefore has important implications for the future potential to limit waste production, a goal that would give priority to prevention.

## Figures and Tables

**Table 1:** Select characteristics of permitted solid waste facilities in North Carolina, 31 December 2003.

					Permit obtained during 1990–2003		
	Total [no. (%)]^d^	Open^a^ [no. (%)]^e^	Public^b^ [no. (%)]^e^	Private^c^ [no. (%)]^e^	Total [no. (%)]^d^	Public^b^ [no. (%)]^f^	Private^c^ [no. (%)]^f^
All types	419 (100)	194 (46)	285 (68)	133 (32)	207 (100)	132 (64)	75 (36)
MSWL	201 (48)	40 (20)	181 (90)	20 (10)	39 (19)	30 (77)	9 (23)
CDLF	75 (18)	63 (84)	58 (77)	17 (23)	73 (35)	57 (78)	16 (22)
TRANSFER	92 (22)	79 (86)	45 (49)	47 (51)	88 (43)	44 (50)	44 (50)
INDUSLF	48 (11)	10 (21)	1 (2)	47 (98)	6 (3)	1 (17)	5 (83)
TIRELF	3 (1)	2 (67)	0 (0)	3 (100)	1 (< 1)	0 (0)	1 (100)

a Accepting waste for disposal or transfer on 31 December 2003.

b Facility owned and operated by public (municipal) entity; owner/operator missing for one facility.

c Facility owned and/or operated by private waste management company or private for special use including military, resort, university, or industrial firm.

d Column percent.

e Row percent.

f Number and row percent for facilities with permits during 1990–2003.

**Table 2:** Associations between presence of a solid waste facility and race and house value: North Carolina block groups, 2003.

		Any solid waste facility		Any MSWL		Any CDLF		Any TRANSFER	
	No.^a^	No. (%)^b^	POR (95% CI)^c^	No. (%)^b^	POR (95% CI)^c^	No. (%)^b^	POR (95% CI)^c^	No. (%)^b^	(95% CI)^c^
All block groups	5,261	251 (4.8)		167 (3.2)		72 (1.4)		84 (1.6)	
Percent people of color									
0 to < 10	1,562	67 (4.3)	1.0^d^	46 (2.9)	1.0^d^	17 (1.1)	1.0^d^	17 (1.1)	1.0^d^
10 to < 20	985	55 (5.6)	1.8 (1.3–2.6)	36 (3.7)	1.9 (1.3–2.8)	14 (1.4)	1.7 (0.9–3.4)	14 (1.4)	1.6 (0.7–3.6)
20 to < 30	647	42 (6.5)	2.5 (1.6–4.0)	30 (4.6)	2.9 (1.8–4.8)	15 (2.3)	3.5 (1.6–7.6)	17 (2.6)	3.3 (1.5–7.5)
30 to < 50	878	43 (4.9)	2.2 (1.4–3.3)	26 (3.0)	2.1 (1.3–3.6)	14 (1.6)	2.9 (1.3–6.4)	18 (2.1)	2.9 (1.4–6.1)
50–100	1,189	44 (3.7)	2.8 (1.9–4.1)	29 (2.4)	2.9 (1.8–4.8)	12 (1.0)	2.7 (1.1–6.4)	18 (1.5)	3.5 (1.7–7.3)
10–100^e^	3,699	184 (5.0)	2.1 (1.6–2.9)	121 (3.3)	2.3 (1.6–3.2)	55 (1.5)	2.4 (1.3–4.5)	67 (1.8)	2.5 (1.3–4.7)
Median house value ($)^f^									
100,000–804,600	1,645	57 (3.5)	1.0^d^	30 (1.8)	1.0^d^	19 (1.2)	1.0^d^	21 (1.3)	1.0^d^
75,000 to < 100,000	1,689	85 (5.0)	1.2 (0.8–1.9)	64 (3.8)	1.4 (0.9–2.3)	27 (1.6)	0.9 (0.4–1.8)	28 (1.7)	1.1 (0.6–2.1)
60,000 to < 75,000	1,105	70 (6.3)	1.8 (1.1–2.8)	47 (4.3)	1.7 (1.0–2.9)	15 (1.4)	0.8 (0.3–1.8)	26 (2.4)	1.5 (0.8–3.0)
0 to < 60,000	822	39 (4.7)	1.5 (0.9–2.5)	26 (3.2)	1.5 (0.8–2.7)	11 (1.3)	0.8 (0.3–2.1)	9 (1.1)	0.8 (0.3–1.8)
0 to < 100,000^g^	3,616	194 (5.4)	1.4 (0.9–2.1)	137 (3.8)	1.5 (0.9–2.4)	53 (1.5)	0.9 (0.4–1.8)	63 (1.7)	1.2 (0.7–2.1)

a Total number of block groups.

b Number of block groups in each category and percent of all block groups with at least one facility.

c Adjusted for population density and region; 95% CI computed with generalized estimating equations using the exchangeable working correlation matrix.

d Referent group.

e Results from a separate model were used to summarize less white block groups and white block groups.

f Median house value for all owner-occupied housing units in US$.

g Results from a separate model to summarize less wealthy block groups to high-wealth block groups.

**Table 3:** Associations between presence of a solid waste facility and block groups classified by race and wealth: North Carolina, 2003.

	0 to < 10% people of color		10 to < 30% people of color		30–100% people of color	
Median house value ($)^a^	No. (%)^b^	POR (95% CI)^c^	No. (%)	POR (95% CI)	No. (%)	POR (95% CI)
100,000–804,600	725 (2.1)	1.0^d^	642 (5.0)	3.8 (1.7–8.3)	278 (3.6)	5.6 (2.5–12.5)
60,000 to < 100,000	775 (6.1)	2.1 (1.2–3.8)	850 (6.5)	3.1 (1.7–5.8)	1,169 (4.5)	4.1 (2.3–7.3)
0 to < 60,000	62 (8.1)	3.1 (1.0–9.6)	140 (7.1)	4.1 (1.9–8.9)	620 (3.9)	3.0 (1.6–5.7)

a Owner-occupied housing units, in US$.

b Block groups in category (percent of all block groups in category with any permitted solid waste facility).

c Adjusted for population density and region, and 95% CI computed with generalized estimating equations using the exchangeable working correlation matrix.

d Referent group.

**Table 4:** Associations between first newly permitted solid waste facility and race and house value, stratified by presence of any previously permitted solid waste facility: North Carolina block groups, 1990–2003.

	No previously permitted facility^a^			Any previously permitted facility^b^		
	Years^c^	No.^d^	HR (95% CI)^e^	Years^c^	No.^d^	HR (95% CI)^f^
Percent people of color						
0 to < 10	23,983	13	1.0^g^	740	28	1.0^g^
10 to < 20	12,846	10	1.6 (0.7–3.6)	545	17	0.8 (0.4–1.7)
20 to < 30	8,346	11	3.0 (1.5–6.1)	380	18	1.1 (0.6–2.2)
30 to < 50	11,111	8	1.8 (0.7–4.3)	322	16	1.1 (0.5–2.5)
50–100	14,135	11	2.7 (1.3–5.7)	388	22	1.0 (0.5–2.1)
10–100^h^	46,438	40	2.2 (1.2–3.8)	1,635	73	1.0 (0.6–1.7)
Median house value ($)^i^						
100,000 to < 787,100	22,350	23	1.0^g^	634	18	1.0^g^
75,000 to < 100,000	22,194	12	0.5 (0.2–0.9)	790	44	1.4 (0.8–2.4)
60,000 to < 75,000	14,735	10	0.6 (0.3–1.6)	583	26	1.3 (0.7–2.4)
0 to < 60,000	11,143	8	0.9 (0.3–2.3)	368	13	1.0 (0.5–2.2)
0 to < 100,000^j^	48,072	30	0.6 (0.3–1.1)	1,741	83	1.3 (0.8–2.2)

a Block group did not contain a permitted solid waste facility eligible to be included in the study prior to 1 January 1990.

b Block group contained a previously permitted solid waste facility included in the study.

c Number of block group years contributed over time at risk for first permitted solid waste facility.

d Number of block groups that received a permitted solid waste facility.

e HR and 95% CI adjusted for population density, computed with robust sandwich estimate.

f HR and 95% CI adjusted for population density, region, and distance to urbanized area/urban cluster, computed with robust variance estimate.

g Referent group.

h Results from a separate model to summarize less white block groups and white block groups.

i Median house value for specified owner-occupied housing units, adjusted for inflation to 2000 US$.

j Results from a separate model to summarize less wealthy block groups and high wealth block groups.

**Table 5:** Associations between first newly permitted solid waste facility and race and house value, by owner/operator: North Carolina block groups, 1990–2003.

		Private solid waste facility^a^		Public solid waste facility^b^	
	Years^c^	No.^d^	HR (95% CI)^e^	No.^d^	HR (95% CI)^e^
Percent people of color					
0 to < 10	24,627	10	1.0^f^	29	1.0^f^
10 to 100	47,813	48	2.4 (1.0–5.8)	59	1.0 (0.6–1.8)
Median house value ($)^g^					
100,000 to < 787,100	22,810	17	1.0^f^	22	1.0^f^
0 to < 100,000	49,630	41	0.9 (0.4–1.9)	66	0.8 (0.5–1.1)

a First permitted solid waste facility in block group is privately owned and/or operated.

b First permitted solid waste facility in block group is publicly owned and operated.

c Number of block-group years contributed over time at risk for first permitted solid waste facility.

d Number of block groups that received a permitted solid waste facility.

e HR and 95% CI adjusted for presence of a permitted solid waste facility in block group before 1 January 1990, population density, distance to nearest urbanized area/urban cluster, distance to nearest major road, and region computed with robust variance estimate.

f Referent group.

g Median house value for specified owner-occupied housing units, adjusted for inflation to 2000 US$.
